# A Mixed Decision Strategy for Freight and Passenger Transportation in Metro Systems

**DOI:** 10.1155/2021/5412016

**Published:** 2021-12-01

**Authors:** Yutao Ye, Junhua Guo, Lixin Yan

**Affiliations:** School of Transportation and Logistics, East China Jiaotong University, Nanchang, Jiangxi Province 330013, China

## Abstract

This paper proposes a mixed decision strategy for freight and passenger transportation in metro systems during off-peak hours (MTS-OPH). The definition of the mixed decision strategy is proposed, and fixed and flexible loading modes are considered for different passenger flow volumes. A mathematical model of the MTS-OPH is proposed and solved using an improved variable neighborhood search algorithm. Case studies demonstrate the performance and applicability of the proposed model and algorithm, and the MTS-OPH is discussed for different delivery distances, passenger flows, and metro network types. The proposed strategy is suitable for long-distance delivery, and the proposed model framework can be applied to different types of metro networks with different levels of complexity. The mixed decision strategy provides a decision support tool for metro and freight companies and can propose corresponding solutions according to different passenger flows.

## 1. Introduction

With the continuous development of e-commerce and home deliveries, urban freight transport has emerged as a key link in urban economic and social development [1, 2]. Road transportation is the primary mode for urban freight transportation [3], and 85–90% of freight is transported by road in France [4]. However, the freight transportation by vehicles poses a series of problems pertaining to urban traffic congestion, greenhouse gas emissions, and noise. Urban freight accounts for 10% of total transportation, but it accounts for 40% of urban pollutant emissions [5]. Therefore, optimizing the transportation structure, strengthening the cooperation of different transportation modes, promoting the organic connection between intercity trunk transportation and city-end distribution [6], and encouraging the development of intensive distribution models are of great significance for creating a green and efficient logistics system.

As mentioned above, the freight transportation needs to change towards more efficient and sustainable transportation systems to cope with increasing demand for freight transportation in urban areas. The metro has the advantages of high efficiency, large capacity, and sustainability, but most metro networks suffer from insufficient utilization of metro trains due to the low passenger flow during off-peak hours [7]. Therefore, introducing goods into the metro network during off-peak hours is a very potential way of freight [8]. The present mixed transport strategy for freight and passenger transportation in metro systems is usually by subjective experience [9], lacking reasonable theoretical framework and mathematical formulation. Furthermore, the entire metro network should be considered to deal with the freight, rather than a single metro line [10]. Therefore, a general theoretical framework and model for constructing the mixed passenger and freight transportation strategy on the metro network during off-peak hours are indispensable.

The purpose of this study is to combine the existing metro network with the first- and last-mile delivery services operated by logistics companies and propose a feasible passenger and freight flow mixed transport strategy for metros during off-peak hours (MTS-OPH). The paper considers fixed and flexible loading modes under different passenger flows and analyzes different cargo characteristics and delivery time requirements. A model for quantitatively evaluating the mixed transportation strategy is established, and an improved variable neighborhood search (VNS) algorithm is designed. They are then applied to the cargo transportation of Ningbo and Beijing metro networks, respectively, and the mixed transportation strategy under different delivery distances, passenger flows, and metro network types is analyzed. The model and method can also be applied to the metro network of other rail transit cities.

The remainder of this paper is organized as follows: Section 2 reviews related research on metro mixed transportation.  Section 3 formulates a nonlinear programing model of the MTS-OPH, and then, an improved VNS algorithm is designed to solve the proposed model in Section 4. Subsequently, two case studies are implemented to verify the proposed model in Section 5. In Section 6, the conclusions of the study and scope for future research are presented.

## 2. Literature Review

### 2.1. Urban Freight and Passenger Transportation

Nash [11] first proposed the use of urban public transportation for freight transportation. Later, based on the concept of sharing, Trentini et al. [12] proposed to introduce urban freight transportation into passenger transportation to achieve the purpose of sharing transportation resources and transportation infrastructure. On this basis, the flow of urban freight and passenger transport was quantified, and a new urban transport system for passenger and freight was constructed and implemented in La Rochelle, France [13]. Gonzalez-Feliu [14] used a socioeconomic cost-benefit analysis to assess the applicability of tram freight transport in the Paris region. The study showed the potential of the tram freight transport mode. Fatnassi et al. [15] integrated personal rapid transportation and freight rapid transportation modes and used electric vehicles to achieve mixed passenger and freight transportation on automatic rails. Masson et al. [16] proposed a hybrid freight method based on the integration of passenger and freight systems to solve mixed urban traffic problems. This method used buses to transport goods from the central distribution center to the transfer point and then used a tricycle to transport the goods from the transfer point to the destination.

There are some successful cases of urban passenger and freight transportation. In Dresden, the tram line from the Volkswagen warehouse to the city center can transport 300,000 tons of goods a year, 10 times a day, which significantly reduces carbon dioxide emissions [17]. In Paris, the commuter line D transports household goods, leisure products, and other goods from the MONOPRIX warehouse (Combs-la-Ville and Lieusaint) on the outskirts of Paris to the Bercy station [18]. These goods are then transported to stores through trucks that use natural gas vehicle fuel to satisfy the emission reduction principles along the logistics line. In New York, subway waste is collected through stations using modified metro trains [19]. However, most of the existing research on mixed transportation modes is based on road and railway transportation. These above studies can be applied into the mixed freight and passenger transportation in the metro system. Since the operation mode, transportation efficiency, and transportation timeliness of metro are different from railways, the mixed transportation of freight and passenger transportation in the metro systems needs further research.

### 2.2. Urban Freight and Passenger Transportation in Metro Systems

The feasibility and application prospects of urban metro freight have been studied in a few research papers. Rijsenbrij and Pielage [20] discussed the feasibility of using the metro for mixed passenger and freight transportation, which gained the attention of national and international scholars on metro freight transportation. Kikuta et al. [9] conducted a test study on the combination of the public metro service and conventional truck operation to prove the feasibility of this mode of transportation. For the metro distribution service, Motraghi and Marinov [21] collaborated with the Newcastle metro network to theoretically analyze urban freight transportation. However, further research is required before actual implementation of their findings. The above research made some explorations on the mixed transportation of freight and passengers in metro systems but did not establish an implementation ability and systematism theory framework. Therefore, a general mixed transportation theoretical framework in the metro systems should be designed to realize the freight and passenger mixed transportation strategy.

In addition, a few studies focused on metro freight. Brice et al. [22] reported a baggage transfer system to facilitate passengers transporting luggage from Newcastle city center to Newcastle International Airport via the metro. They proved the feasibility of the new baggage transfer solution and reported that the corresponding cost is higher than that of the existing service. Ghilas et al. [23] found that integrated freight and scheduled line services can potentially reduce the operating costs of logistics service providers and the public transportation sector can obtain additional revenue. However, the existing mixed transportation models are established based on a single metro line. Considering the different directions of goods circulation, existing models cannot accurately describe the mixed transport strategy in metro systems. Therefore, a mixed transportation model on the metro network should be established to realize the circulation of goods.

### 2.3. Optimization Method of Urban Freight and Passenger Transportation

Location and route selection have long been the significant issues in the freight and passenger transportation domain. Fatnassi et al. [15] proposed two mathematical formulas to solve the vehicle route between stations and used dynamic optimization methods and developed algorithms to solve the shared transportation of goods and passengers. Zhao et al. [24] proposed a segmentation method of urban metro network, using complex network theory and the TOPSIS model to determine the candidate metro distribution hubs for the location model. Dong et al. [25] analyzed the characteristics of underground cargo capacity and established a mixed integer programing model to select the location of metro distribution hubs. However, the limitation of the frequency of goods transfer in the metro network is barely considered in these studies.

Likewise, time window constraint is a critical issue along with the rapid increase in freight demand during recent years. Behiri et al. [8] studied an environment-friendly urban freight transportation alternative using a passenger railway network and proposed a heuristic based on dispatching rules and a single-train-based decomposition heuristic to solve the Freight-Rail-Transport-Scheduling Problem. Yang et al. [26] studied the vehicle routing problem with mixed backhauls and time windows for city logistics, and the time-dependent pickups and deliveries can be depicted by extending the state dimensions. However, freight transportation in the passenger transportation system focuses on the study of the cargo time window, ignoring the impact on the normal operation time window of the passenger transportation system.

In addition, the development of solving algorithms has attracted the attention of researchers and practitioners. Bräysy [27] introduced the internal design of variable neighborhood descent and variable neighborhood search algorithms in detail, analyzed the vehicle routing problem with time windows problem, and pointed out that the variable neighborhood search algorithm is one of the most effective methods to solve the vehicle routing problem with time windows problem. de Armas and Melián-Batista [28] studied a dynamic rich vehicle routing problem with time windows and proposed a meta-heuristic algorithm based on variable neighborhood search to solve this problem. However, the stability and reliability of the calculation results need to be strengthened. Therefore, we design two types of neighborhood structures to obtain high-quality solutions.

### 2.4. Contribution

The main contributions of this study are summarized as follows:

- We propose a general theoretical framework of MTS-OPH. This framework includes new concept and transportation standards of MTS-OPH. This definition clarifies the process and applicable time of mixed transportation. Relative to previous study on the types of mixed transportation goods [13, 29], MTS-OPH further explored the types of goods suitable for mixed transportation on the basis of transporting small goods. The mixed transportation standard of separate loading of passengers and cargo and priority transportation of passenger flow is established. Furthermore, fixed and flexible cargo loading modes are proposed according to different off-peak passenger flows.
- We formulate a nonlinear programing model for the MTS-OPH. Based on the research of Fatnassi et al. [15] and Zhao et al. [30], our model improves the mixed transportation strategy research to a mixed transportation strategy research under load rates of passenger flow during off-peak hours. To the best of our knowledge, this is the first time that the problem of mixed passenger and freight transportation under different passenger flows has been addressed in the context of metro transportation during off-peak hours. Moreover, an improved VNS algorithm is designed to solve the model, which provides a decision support tool for logistics companies.
- We presented the real case study of the Ningbo and Beijing metro network to verify the practicality and efficiency of the proposed model. The applicability of the model is discussed from the distribution distance, off-peak passenger flow, and metro network type. Our results show that the proposed model can be applied to different metro networks. Furthermore, the mixed transportation mode in the metro systems has the advantages of high speed, high punctuality, low economic investment, low environmental impact, and low energy consumption [31–33].

## 3. Mathematical Formulation

This section provides a detailed description of the MTS-OPH, analyzes the types of goods suitable for mixed transportation, and provides different mixed transportation methods based on different load rates of passenger flow during off-peak hours. Finally, a nonlinear programing model of a mixed transportation strategy is constructed.

### 3.1. Description of MTS-OPH

**Figure 1:**
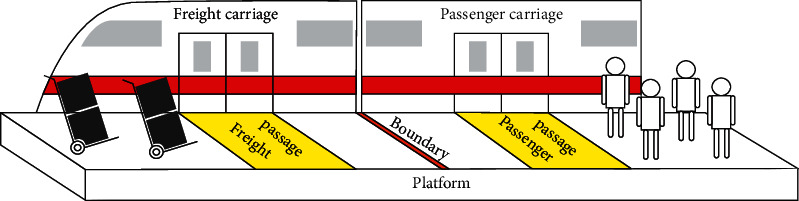
Processing of the second stage.

MTS-OPH integrates the existing urban metro network with the first- and last-mile delivery services operated by logistics firms. The key aspect of this strategy is to transport goods and passengers together without affecting the metro passenger flow. Thus, the transportation standard of separate loading of passengers and cargo and priority transportation of passenger flow should be considered. First, metro train carriages are divided into passenger carriages and freight carriages, similar to the passenger carriages and female carriages set in the Shenzhen metro trains in China [34]. Different types of carriages are set with boundary lines and dedicated passages to divide the passenger and freight flows, as shown in Figure 1. To minimize the impact of cargo flow on passengers, we introduce relevant constraints on the types of cargo used for mixed transportation. For ease of operation, all goods are placed in a same-size freight box as freight parcels. Concurrently, small cargo packages should be selected for metro mixed transportation. A similar conclusion has been reported in related studies on bus freight [13] and metro freight [9, 29]. Thus, goods in the freight parcel should be small goods, such as documents, books, clothing, and small mechanical parts, rather than fresh goods that need to be frozen and kept fresh, which are not affected by time and the environment, in order to maintain the metro carriage environment tidy.

**Figure 2:**
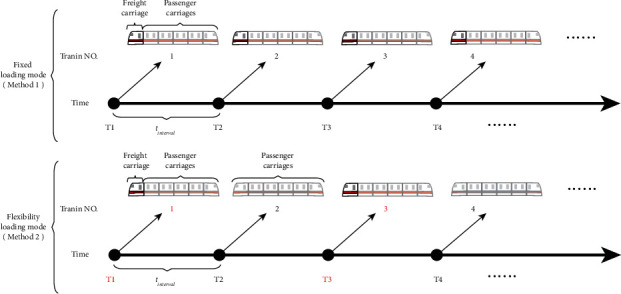
Two transportation methods of MTS-OPH.

Second, to satisfy the passenger flow priority transportation standard without changing the headway and stop time of metro trains during off-peak hours, this study proposes different mixed transportation methods based on different load rates of passenger flow during off-peak hours. When the number of passengers and freight demand is greater than the metro capacity, each train adopts a mixed passenger and cargo transportation mode during off-peak hours, as shown in Method 1 (i.e., fixed loading mode) in Figure 2. When the number of passengers and freight demand is less than the metro capacity, one of every two trains is selected to employ the mixed transportation mode while the other train is used for passenger transportation, as shown in Method 2 (i.e., flexibility loading mode) in Figure 2. In Figure 2, *t*_interval_ represents the headway of metro trains during off-peak hours.

**Figure 3:**
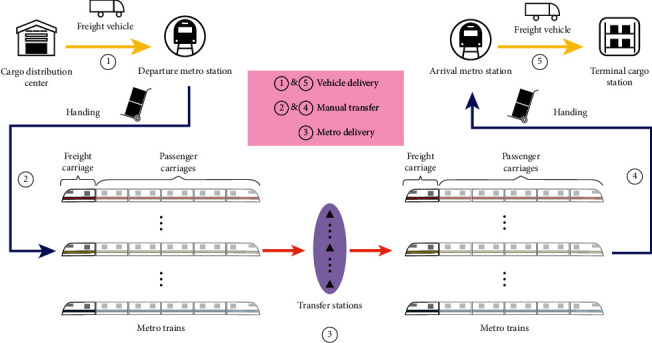
Operation process of MTS-OPH.

In summary, MTS-OPH is a transportation strategy based on the aforementioned mixed transportation standard, which satisfies the optimal distribution cost of the cargo flow under the condition of determining the origin and terminal stations of the goods. The transportation strategy is to use the metro passenger transportation network, which is composed of cargo distribution centers, metro stations, terminal cargo stations, metro trains, and freight vehicles, as shown in Figure 3.

In Figure 3, the operation process of MTS-OPH is divided into five stages. The first and fifth stages are vehicle delivery, second and fourth stages are manual transshipments, which transfer cargo from the vehicle to metro carriage, and third stage is metro delivery. The transfer of goods between each stage is completed by trolley.

In the first stage, the cargo is packed in freight parcels, placed in freight boxes, loaded onto transportation vehicles, and delivered to the departure metro station via freight vehicles. Then, the freight boxes are unloaded from the vehicle and loaded on the trolley. In the second stage, the freight boxes are transported to the metro platform via trolleys and transported to the metro freight carriage when the train enters the platform. In the third stage, freight boxes are transported on the metro network (transfers are performed via trolleys). Here, the train operation mode is from the first station to the last station regardless of the train service route. The freight boxes enter the fourth stage after being transported via the metro train. The freight boxes are unloaded from the metro freight carriage and transported via trolleys to the arrival metro station. In the last stage, the freight boxes are loaded onto the freight vehicle and transported to the corresponding terminal cargo station. All the aforementioned stages constitute the MTS-OPH operation process, while the third stage of the operation period in MTS-OPH is the off-peak hours of metro operation.

### 3.2. Assumptions and Notations

The MTS-OPH model can be described as the process of delivering multiple freight stations from a cargo distribution center with the optimal delivery cost as the goal. The number of terminal cargo stations is determined, but the freight stations are located in different geographical locations. All transportation paths are based on the actual shortest distance of the road network or metro network. Thus, certain assumptions have been considered as follows:

- The freight is placed in a standardized unit parcel to measure the arrival demand of the terminal cargo stations.
- There is no storage function at the metro exit station, and freight vehicles are arranged for delivery immediately after the cargo arrives at the metro exit station.
- The freight vehicles used for transportation are of the same model with the same fuel consumption and load capacity.
- The freight vehicles run at a uniform velocity without consideration of the road conditions. Furthermore, after the freight is transported, the vehicles are not required to return to the cargo distribution center or arrival metro stations.
- Up to two freight transfers occur in the urban metro network.

Table 1 summarizes the notations used throughout the paper.

### 3.3. System Constraints

#### 3.3.1. Number of Freight Carriages Constraints

(1) $$\begin{array}{c} E=D-\left[\frac{\max N}{{\text{cap}}_{M}}\right],m=1,\ldots ,M. \end{array}$$

Equation (1) determines the number of freight carriages ((*x*) represents the smallest integer greater than *x*).

#### 3.3.2. Freight Loading Mode Constraints

(2) $$\begin{array}{c} r=\left\{\begin{array}{c} 1,\;\frac{Q_{i^{\prime }}}{E\times {\text{cap}}_{M}}>1,\\ 2,\;\frac{Q_{i^{\prime }}}{E\times {\text{cap}}_{M}}\leq 1, \end{array}\right.\;i^{\prime }=1,\ldots ,I^{\prime }. \end{array}$$

The freight loading mode is selected by Equation (2). Among them, *r*=1 is a fixed loading mode; that is, each train uses a mixed passenger and freight transportation mode during off-peak hours; *r*=2 is a flexible loading mode; that is, one of every two trains chooses to use a mixed transportation mode and the other for passenger transportation.

#### 3.3.3. Vehicle Line Capacity Constraints

Vehicles in the “first mile and last-mile” distribution network should meet vehicle capacity constraints, vehicle number constraints, and line capacity constraints.

(3) $$\begin{array}{c} \overset{n}{\underset{v=1}{\sum }}x_{ij}^{v}=1,\;i=1,j=1,\ldots ,J, \end{array}$$

(4) $$\begin{array}{c} \overset{V}{\underset{v=n+1}{\sum }}\overset{J^{\prime }}{\underset{j^{\prime }=1}{\sum }}x_{j^{\prime }i^{\prime }}^{v}=1,\;i^{\prime }=1,\ldots ,I^{\prime }, \end{array}$$

(5) $$\begin{array}{c} Q_{i^{\prime }}\leq \overset{V}{\underset{v=n+1}{\sum }}\overset{J^{\prime }}{\underset{j^{\prime }=1}{\sum }}x_{j^{\prime }i^{\prime }}^{v}{\text{cap}}_{V}\text{,}\;i^{\prime }=1,\ldots ,I^{\prime }, \end{array}$$

(6) $$\begin{array}{c} \overset{J}{\underset{j=1}{\sum }}\overset{n}{\underset{v=1}{\sum }}x_{ij}^{v}+\overset{J^{\prime }}{\underset{j^{\prime }=1}{\sum }}\overset{I^{\prime }}{\underset{i^{\prime }=1}{\sum }}\overset{V}{\underset{v=n+1}{\sum }}x_{j^{\prime }i^{\prime }}^{v}=K,\;i=1. \end{array}$$

Equation (3) assigns a unique route for each vehicle from the cargo distribution center to the departure metro station. Equation (4) imposes the restriction that each vehicle can have one unique route from one arrival metro station to one terminal cargo station in the third stage. Equation (5) constrains the capacity on the route. The total number of vehicles in the complete distribution process should be restrained, as shown in Equation (6).

#### 3.3.4. Metro Line Capacity Constraints

The goods in the metro network by train should meet the train capacity constraint and the number of transfer constraints.

(7) $$\begin{array}{c} Q_{i^{\prime }}\leq E\times {\text{cap}}_{M}\times \overset{M}{\underset{m=1}{\sum }}\overset{J}{\underset{j=1}{\sum }}\overset{J^{\prime }}{\underset{j^{\prime }=1}{\sum }}y_{jj^{\prime }}^{i^{\prime }m},\;i^{\prime }=1,\ldots ,I, \end{array}$$

(8) $$\begin{array}{c} \overset{J}{\underset{j=1}{\sum }}O_{jj^{\prime }}\leq 2,\;j^{\prime }=1,\ldots ,J^{\prime }. \end{array}$$

Equation (7) expresses the capacity limit of the metro. Equation (8) stipulates the number of transfers in the metro network.

#### 3.3.5. Delivery Time Constraint

The mixed transportation is based on the off-peak hours of urban rail transit, and the departure time of mixed transportation trains shall not be earlier than the start time of off-peak hours.

(9) $$\begin{array}{c} \overset{j-1}{\underset{s=1}{\sum }}t_{s}+s_{j}^{m}\leq t_{j},\;j=1,\ldots ,J,m=1,\ldots ,M, \end{array}$$

(10) $$\begin{array}{c} t_{j}-t_{ij}-\left(1-x_{ij}^{v}\right)Z\leq {\text{SET}}_{i^{\prime }},\;i=1,j=1,\ldots ,J,v=1,\ldots ,n,i^{\prime }=1,\ldots ,I^{\prime }, \end{array}$$

(11) $$\begin{array}{c} {\text{ET}}_{i\prime }\leq {\text{SET}}_{i^{\prime }}\leq {\text{LT}}_{i^{\prime }},\;i^{\prime }=1,\ldots ,I^{\prime }. \end{array}$$

Equation (9) determines the earliest time when the freight to cargo station is loaded into the train at a station. In this equation, *t*_*s*_ represents the running time between *S* and *S *−* *1 when the train is at station *S*, and when *s*=1, *t*_*s*_=0. Equation (10) ensures the time to start delivery for the terminal cargo station. Equation (11) is the time window constraint of the terminal cargo station.

The delivery time of the train in the whole mixed transportation process meets the sum of the delivery time of the “first mile and last-mile” and the metro delivery time.

(12) $$\begin{array}{c} {\text{SLT}}_{i^{\prime }}={\text{SET}}_{i^{\prime }}+t_{ij}+\left(t_{jj^{\prime }}+r\times t_{\text{interval}}\right)y_{jj^{\prime }}^{i^{\prime }m}+O_{jj^{\prime }}t_{O}+t_{j^{\prime }i^{\prime }},\;j=1,\ldots ,J,\\ j^{\prime }=1,\ldots ,J^{\prime },i^{\prime }=1,\ldots ,I^{\prime },m=1,\ldots ,M. \end{array}$$

Equation (12) addresses the relationship between the start time and end time of delivery from the cargo distribution center to the terminal cargo station.

The delay cost coefficient is affected by the delivery time window of the goods. Failure to complete the delivery within the time window needs to calculate the delay cost based on the delay cost coefficient.

(13) $$\begin{array}{c} \lambda_{i^{\prime }}=\left\{\begin{array}{c} 0,\;{\text{SLT}}_{i^{\prime }}\leq {\text{LT}}_{i^{\prime }},\\ \alpha ,\;{\text{SLT}}_{i^{\prime }}\geq {\text{LT}}_{i^{\prime }}, \end{array}\right.\;i^{\prime }=1,\ldots ,I^{\prime }. \end{array}$$

Equation (13) addresses the delay cost due to the failure to complete the delivery within the time window; the delay cost is related to the time of completion of the delivery.

#### 3.3.6. Decision Variable Constraints

The relevant decision variables are constrained as follows. Equation (14) expresses the constraint on the initial number of vehicles.

(14) $$\begin{array}{c} K\geq 2, \end{array}$$

(15) $$\begin{array}{c} {\text{SET}}_{i^{\prime }},{\text{SLT}}_{i^{\prime }}\geq 0,\;i^{\prime }=1,\ldots ,I^{\prime }, \end{array}$$

(16) $$\begin{array}{c} x_{ij}^{v}\in \left\{0,1\right\},\;i=1,\ldots ,I,j=1,\ldots ,J,v=1,\ldots ,n, \end{array}$$

(17) $$\begin{array}{c} x_{j^{\prime }i^{\prime }}^{v}\in \left\{0,1\right\},\;\;j^{\prime }=1,\ldots ,J^{\prime },i^{\prime }=1,\ldots ,J^{\prime },v=n+1,\ldots ,V, \end{array}$$

(18) $$\begin{array}{c} y_{jj^{\prime }}^{i^{\prime }m}\in \left\{0,1\right\}\text{,}\;\;j=1,\ldots ,J,j^{\prime }=1,\ldots ,J^{\prime },i^{\prime }=1,\ldots ,I^{\prime },m=1,\ldots ,M. \end{array}$$

### 3.4. Composition of the Objective Function

The objective function of MTS-OPH is composed of vehicle transportation cost, transfer cost, and delay cost. Each cost parameter is described in the following.

#### 3.4.1. Vehicle Transportation Cost

The transportation cost of vehicles is mainly composed of the transportation distance, number of vehicles, number of freight parcels, and fixed operating cost of vehicles. Therefore, the transportation cost *f*_*vij*_ in route (*i*, *j*) is as shown in

(19) $$\begin{array}{c} f_{vij}=Q_{i^{\prime }}y_{jj^{\prime }}^{i^{\prime }m}x_{ij}^{v}C_{ij}. \end{array}$$

To express *f*_*vij*_ clearly, Equation (19) is simplified as

(20) $$\begin{array}{c} Q_{ij}=Q_{i^{\prime }}y_{jj^{\prime }}^{i^{\prime }m}x_{ij}^{v}, \end{array}$$

(21) $$\begin{array}{c} f_{vij}=C_{ij}Q_{ij}. \end{array}$$

Therefore, the vehicle transportation cost *f*_*vj*′*i*′_ in the route (*j*′, *i*′) can be obtained similarly.

(22) $$\begin{array}{c} f_{vj^{\prime }i^{\prime }}=C_{j\prime i\prime }Q_{i\prime }. \end{array}$$

In summary, Equation (23) represents the vehicle transportation cost.

(23) $$\begin{array}{c} f_{v}=\sum_{i=1}\sum_{j=1}^{J}\sum_{v=1}^{n}C_{ij}Q_{ij}x_{ij}^{v}+\sum_{j=1}^{J^{;}}\sum_{i^{\prime }=1}^{I^{\prime }}\sum_{v=n+1}^{V}C_{j^{\prime }i^{\prime }}Q_{i^{\prime }}x_{j^{\prime }i^{\prime }}^{v}+KC_{v}. \end{array}$$

#### 3.4.2. Transfer Cost

The number of freight parcels and frequency of transfer constitute the transit cost. The number of transfers is mainly determined by the number of times the cargo is loaded and unloaded; the completion of one loading and unloading of cargo is regarded as one transfer. Equation (24) shows the calculation method for the node transfer cost *f*_*n*_.

(24) $$\begin{array}{c} f_{n}=2C_{O}\left(\sum_{i^{\prime }=1}^{I^{\prime }}Q_{i^{\prime }}+\sum_{j=1}^{J}\sum_{j^{\prime }=1}^{J^{\prime }}\sum_{i^{\prime }=1}^{I^{\prime }}\sum_{m=1}^{M}O_{jj^{\prime }}y_{jj^{\prime }}^{i^{\prime }m}Q_{i^{\prime }}\right). \end{array}$$

#### 3.4.3. Delay Cost

The delay cost is caused by the freight arriving at the terminal cargo station in an unexpected time window. The delivery time window of MTS-OPH is based on the off-peak start time, so the delay cost will only be calculated if the delivery time window is exceeded. This is expressed with *f*_*d*_, as in

(25) $$\begin{array}{c} f_{d}=\sum_{i^{\prime }=1}^{I^{\prime }}\lambda_{i^{\prime }}Q_{i^{\prime }}. \end{array}$$

Because only part of the metro carriages is used for freight, the weight of the cargo is much smaller than that of the metro. Therefore, the delivery cost of MTS-OPH is not affected by metro fixed operating cost and metro transportation distance.

In summary, the delivery cost *f*_*C*_ is the sum of the vehicle transportation cost *f*_*v*_, node transit cost *f*_*n*_, and delay cost *f*_*d*_, i.e., *f*_*C*_=*f*_*v*_+*f*_*n*_+*f*_*d*_. Hence, the mathematical formulation of the MTS-OPH model is built as follows:

(26) $$\begin{array}{c} \min Z=f_{c}. \end{array}$$

Equation (26) denotes the smallest delivery cost, and the calculation process is, respectively, performed using Equations (19)–(25).

In the MTS-OPH model, the pickup and delivery constraints of the time window and transshipment value are considered [8, 35, 36] and the mode selection constraints are added according to the two loading modes of the proposed mixed transport strategy. This model is developed based on the generalized assignment problem, which belongs to a NP-hard problem [37–39] and is usually solved by heuristic algorithms.

## 4. Solution Approaches

The heuristic algorithm for solving the MTS-OPH is based on the VNS [40, 41]. VNS provides a flexible framework for constructing heuristics for approximately solving combinatorial optimization problems and nonlinear optimization problems. The main idea is to dynamically change the neighborhood structure set during the search process to expand the search range and obtain local optimal solutions. Using such a variable neighborhood strategy, it is possible to move away from the optimum and finally reach convergence after multiple iterations. In the VNS algorithm used in this study, the objective function (indicated by *Z*_*c*_) in Equation (8) is used as an evaluation index for evaluating the quality of the generated solution, as shown in the following:

(27) $$\begin{array}{c} f\left(c\right)=Z_{c}. \end{array}$$

In this study, the VNS algorithm includes the following three parts: initial solution, shaking process, and variable neighborhood descent (VND) process. We use *c*, *c*′,  and *c*^″^ to denote the solutions generated in the algorithm.*G* represents the neighborhood structure set included in the shaking process, where *G*={1,2,3,…, *p*,…, |*G*|}, and *H* represents the neighborhood structure set included in the VND process, where *H*={1,2,3,…, *p*,…, |*H*|}. The detailed structure of the improved VNS algorithm is shown in Algorithm 1.

The shaking process is a perturbation operator in the VNS. The process is used to generate different neighborhood solutions. The remaining initial solution and VND process are described in detail in the next section.

### 4.1. Initial Solution

**Figure 4:**
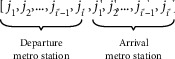
Initial path diagram.

The initial delivery route *R* of the MTS-OPH is formed by constructing a line of length 2*I*′ according to the number *i*′ of a terminal cargo station. The initial route *R* is mainly composed of *i*′ departure metro stations and *i*′ arrival metro stations shown in Figure 4. Herein, each *j*_*i*′_ in [*j*_1_, *j*_2_,…, *j*_*i*′−1_, *j*_*i*′_] corresponds to each *j*_*i*′_′ in [*j*_1_′, *j*_2_′,…, *j*_*i*′−1_′, *j*_*i*′_′] individually, and a group [*j*_*i*′_, *j*_*i*′_′] constitutes a metro distribution route. The following conditions should be met: *j* ∈ *J*, *j*′ ∈ *J*′,  and *J*=*J*′.

### 4.2. Neighborhood Structure

Two types of neighborhood structures are applied. The first consists of neighbors which exchange strategies on existing delivery routes. The second consists of neighbors obtained by updating the distribution strategy on the existing route. The methods for generating neighbors for the first type of neighborhood structure are Swap-2 and Inserting-t [42]. For the second type of neighborhood structure, Alter-t method of producing neighbors is designed in our study.

**Figure 5:**
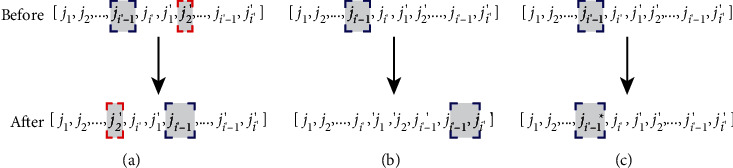
Three methods of producing neighbors. (a) Swap-2. (b) Insertion-1. (c) Alter-1.

Swap-2 refers to the random exchange of two adjacent or nonadjacent rows in the initial solution, as shown in Figure 5(a). Insertion-t is formed by repeating *t* times on the basis of Insertion-1. As shown in Figure 5(b), Insertion-1 randomly deletes a row from the initial solution and randomly inserts it into other positions. In Figure 5(c), Alter-1 randomly selects a position *j*_*i*′_ in the initial solution and selects a new number from the set *J* to replace *j*_*i*′_. Alter-t is to repeat the Alter-1 operation *t* times. In addition, to prevent the value of *t* from being too large and destroying the stability of the obtained solution structure, the value of *t* is controlled to be in the range of [0, 5] in the insertion operation and change operation of this study. The iterations of repeated deletions, changes, and insertions let the algorithm search in a larger solution space, thereby enhancing the ability of the neighborhood search algorithm to move away from the local optimal region.

### 4.3. Shaking Procedure

We use *G* = {Alter−3, Alter−4} as the set of neighborhood structures in the shaking procedure. For each structure *G*_*v*_ ∈ *G*, it maps a given solution *r* to a series of neighborhoods *G*_*v*_(*c*). When the shaking procedure is applied, a solution will be randomly chosen from the neighborhoods. Accordingly, the detailed procedure is given in Algorithm 2.

### 4.4. Variable Neighborhood Descent

During a local search in VND, when a better solution than the current solution cannot be found in this neighborhood, the search is continued by moving to the next neighborhood solution. Contrarily, if a better solution than the current solution is found in this neighborhood, the first neighborhood solution will be returned to restart the search. For a better solution, the first neighborhood solution should be returned and the search should be started again. The local optimal solution obtained through such a search process is likely to be the global optimal solution. A detailed operation of the VND process is provided in Algorithm 3.

## 5. Numerical Experiments

Case studies of the Ningbo metro network and Beijing metro network were conducted to evaluate the accuracy and efficiency of the proposed model and method. Examples of the application in different delivery distances, different passenger flows, and different types of metro networks were illustrated. The proposed algorithm framework was coded in MATLAB 10.0 on a Window 10 personal computer with 4.0 GB processor. The MTS-OPH problem is solved by IBM CPLEX 12.5 Academic Version on the same platform.

### 5.1. Small-Scale Case Study

**Figure 6:**
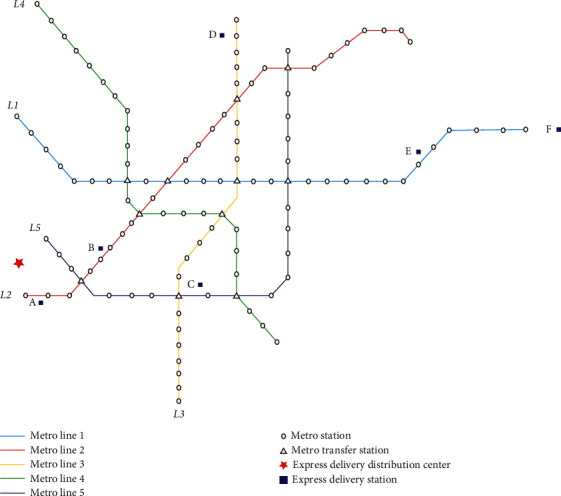
Ningbo metro network schematic diagram.

In this section, based on the logistics information of a certain express company in Ningbo, we considered a distribution situation between the express delivery distribution center and six terminal express delivery stations. For convenience, the cargo distribution center is represented by *I* and the six cargo stations are named A, B, C, D, E, and F, respectively, i.e., *i*′={1,2,3,4,5,6}, *i*′ ∈ *I*′. The geographical location of freight stations is evenly distributed, and the receipt volume of each cargo station obeys the uniform distribution of [40, 60]. The metro network of the Ningbo Urban Rapid Rail Transit Construction Plan (2013−2020) [43] was considered as an example, as shown in Figure 6.

The airport logistics park near the metro line was selected as the express delivery distribution center, which is represented by a red star in Figure 6. L1−L5 indicate each metro line. Considering the actual situation and model solution, each metro line station was uniformly numbered according to positive integers from left to right, with the letter “I” representing the departure metro station and “O” representing the arrival metro station.

The average speed of freight vehicles was 20 km/h [30], and the delivery cost of unit express delivery per unit distance of freight vehicles was assumed to be 2 yuan [44]. The values of the other parameters are listed in Table 2. In the experiment, the distribution data and time window are shown in Table 3.

In the algorithm parameter setting process, this study sets the number of neighborhood solutions in one iteration to 100 and the total number of iterations to 50 [27]. The neighborhood structures of VND and shaking are as follows: VND, {Alter_3, Alter_5, Insertion_2, Insertion_1, Opt_2}; Shaking, {Alter_3, Alter_5}.

For related problems in route planning and distribution, the genetic algorithm (GA) is widely used [45–47]. Therefore, the GA and the improved VNS algorithm are used to solve the problem. The population size of the GA is 100, the number of iterations is 50, the crossover probability is 0.6, and the mutation probability is 0.1 [48]. The optimization results of the two algorithms were obtained by running 20 times, as shown in Table 4. Although GA has a shorter calculating time, VNS can obtain the best delivery cost of higher quality, and after many repeated trials, the results show that the stability of the optimal solution obtained by VNS is better than that of GA, which verifies the effectiveness of the algorithm.

In order to further prove the effectiveness of the improved VNS algorithm, we first give the actual maximum cross-section passenger flow to Equation (12), making MTS-OPH an integer linear programing model. Secondly, the ILOG CPLEX solver is used to solve it. After implementations, we finally obtained the returned solution with a calculation time of 328 s, where the relative gap turns out to be 5.00%. The approximate optimal objective value is 5861.0 yuan, which is consistent with the solution result of the improved VNS algorithm. Therefore, the proposed algorithm is effective.

Finally, the optimal solution for the delivery of vehicles is calculated. Meanwhile, the optimal transportation strategy during off-peak hours for the individual distribution of freight vehicles is calculated; this transportation strategy is called VTS-OPH.  Table 5 shows the comparison between the MTS-OPH and VTS-OPH. The delivery time of VTS is mainly composed of two parts: loading and unloading time and transportation time. Among them, the loading and unloading time is determined according to the quantity of goods and the transportation time is determined by the transportation distance and the transportation speed. If the delivery time is less than 1 min, it is approximated to 1 min.

As evident from Table 5, the MTS-OPH and VTS-OPH complete deliveries within the time window. The average time to complete a delivery in the VTS-OPH is 69 min, and in the MTS-OPH, the average time is 73 min. However, the MTS-OPH spends approximately one-third of the delivery cost of the VTS-OPH to complete a delivery. According to this small-scale case analysis results, the MTS-OPH has certain advantages when compared with the VTS-OPH.

The delivery route is shown in Table 6, and the result shows that for the express station “F” delivery, the MTS-OPH requires less time than the VTS-OPH, and for the delivery of the other express stations, the VTS-OPH requires less time than the MTS-OPH. Therefore, further investigation is required regarding the applicability of the MTS-OPH for different distances.

### 5.2. Applicability of MTS-OPH considering Different Delivery Distances

**Figure 7:**
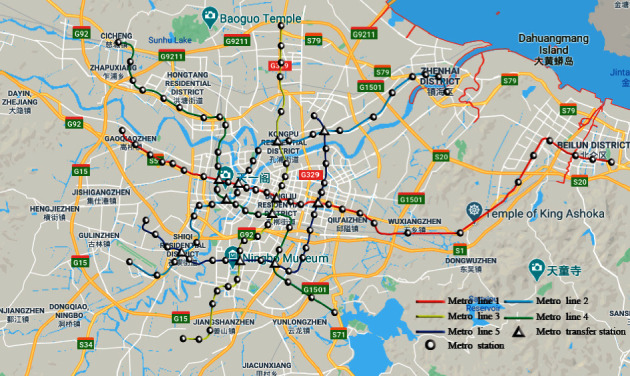
Metro network of Ningbo in 2020.

To study the applicability of the MTS-OPH for different delivery distances, we divide the actual express delivery information provided by the airport logistics park into three different distribution range data tables. The metro network of Ningbo is shown in Figure 7.

**Figure 8:**
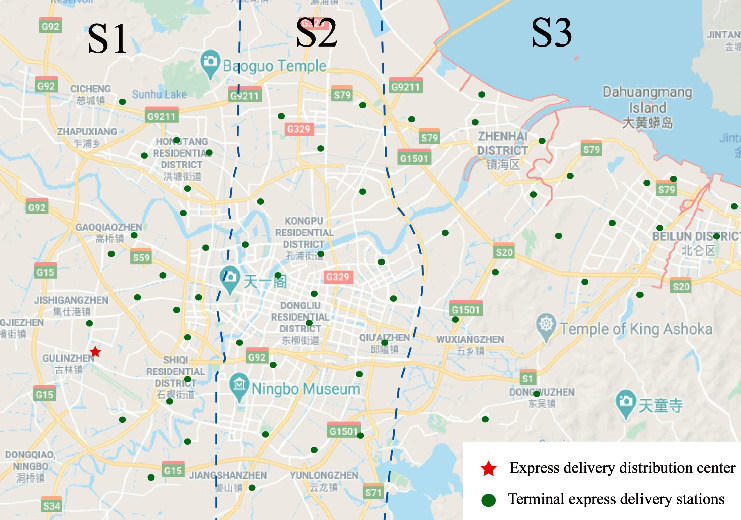
Distribution area.

According to the metro network and express delivery information data, Ningbo is divided into three areas with different distribution scopes as S1, S2, and S3. Twenty delivery destinations (terminal express delivery stations) are selected with uniform locations in each area, as shown in Figure 8. Each delivery area is separated by a blue dotted line, where S1 is a short-distance delivery area, S2 is a medium-distance delivery area, and S3 is a long-distance delivery area. According to different distribution ranges, different time window requirements are allotted [44], as shown in Table 7.

In the experiment, the time horizon is considered to be 9:00−12:00, which is the off-peak period of metro operation. In addition, the metro model in Ningbo is type B composed of six carriages with a total capacity of 1,460 people. According to statistics from the Ningbo Rail Transit Group, the maximum load rate during off-peak hours in the Ningbo metro network is 90%. Hence, at least five metro carriages are required for passenger transportation during off-peak hours, and the remaining one carriage is used for goods transportation. The remaining parameter values and algorithm parameter settings are consistent with the small-scale case study presented in Section 5.1.

#### 5.2.1. Analysis of the Short-Distance Delivery Area

The demand and time window of the terminal express station in S1 are listed in Table 8.

The data in Table 8 are used in the VNS algorithm for 20 calculations to obtain the optimal solution for the MTS-OPH and VTS-OPH, as shown in Table 9, and the optimal delivery routes of the two strategies are given in Table 10.

In Table 9, the delivery cost of the MTS-OPH is approximately 66.4% higher than that of the VTS-OPH. In addition, 12 of the 20 terminal express delivery stations in the MTS-OPH failed to deliver on time, while the VTS-OPH completed all deliveries with the set time window. In the short-distance distribution area, the success rate of the MTS-OPH to complete the delivery within the time window was 40%. However, the MTS-OPH reduces the vehicle delivery distance by 56.7% when compared with that of the VTS-OPH. Although the vehicle transportation distance is reduced, the delivery time is increased, as shown in Table 10. According to the above analysis, in the short-distance delivery area, the MTS-OPH is not suitable for multitarget delivery and the overall operation of express delivery companies, but the single-target delivery remains to be studied. This corresponds well with the study of high-speed railway freight distribution by Pazour et al. [49].

#### 5.2.2. Analysis of the Medium-Distance Delivery Area

The demand and time window of the terminal express station in S2 are listed in Table 11.

Similarly, the data in Table 11 are used in the VNS algorithm for 20 calculations, and the optimal solution is listed in Table 12.

As shown in Table 12, the delivery cost of the VTS-OPH is 8.0% higher than that of the MTS-OPH, but the average delivery time of the VTS-OPH is 33.7% less than that of the MTS-OPH. In addition, the specific delivery time, delivery route, transfer times, and train number of each express station under the two modes are shown in Table 13. Concurrently, VTS-OPH completed all deliveries within the time window. There were two express delivery stations in the MTS-OPH that failed to deliver on time. The vehicle delivery distance in MTS-OPH is 24.7% of that in VTS-OPH.

Combining the results listed in Tables 9 and 12, the number of express stations that did not deliver on time significantly improved when the MTS-OPH was selected in the medium-distance delivery area, when compared with the MTS-OPH in the short-distance delivery area. From the original 12 express stations that failed to deliver on time, two express delivery stations failed to deliver on time. Therefore, although the VTS-OPH requires less time to complete 100% on-time distribution, the MTS-OPH delivery cost is slightly lower and the MTS-OPH significantly reduces the vehicle transportation distance. This means that when choosing the MTS-OPH for delivery, the transportation distance of vehicles can be reduced and pressure on urban roads can be reduced. According to the above analysis, the MTS-OPH is more suitable for the medium-distance distribution area than for the short-distance distribution area. The MTS-OPH and VTS-OPH exhibit varied advantages in medium-distance delivery areas; however, considering the urban system, the MTS-OPH is better than the VTS-OPH.

#### 5.2.3. Analysis of the Long-Distance Delivery Area

The demand and time window of the terminal express station in S3 are shown in Table 14.

According to the above experimental data, the VNS algorithm is used to solve the two transportation strategies of MTS-OPH and VTS-OPH 20 times and the optimal solution obtained is shown in Table 15. The delivery route information is shown in Table 16.

As shown in Tables 15 and 16, we obtained a different delivery result when compared with results of previous analyses. In the long-distance delivery area, all indicators of the MTS-OPH are better than those of the VTS-OPH, except for the average delivery time. The delivery cost of MTS-OPH is 52.2% of VTS-OPH, the vehicle delivery distance is 17.6% of VTS-OPH, and the average delivery time is slightly worse than that of VTS-OPH. A significant change is observed in the number of express delivery stations that failed to deliver on time. In the MTS-OPH, all express delivery stations completed the delivery within the time window, while in the VED mode, there were four express stations that failed to complete the delivery within the time window. Thus, the MTS-OPH is suitable for long-distance distribution areas. A similar result was reported in a high-speed rail express delivery study conducted in [50].

Concurrently, by comparing the calculation results in Tables 9, 12, and 15, it is further verified that the MTS-OPH is suitable for long-distance delivery. On the one hand, as the distribution distance increased, the distribution cost of the MTS-OPH changed slightly and the vehicle delivery distance increased at an average growth rate of 18%, which was much smaller than that of the VTS-OPH and increased with an average growth rate of 86%. The above analysis proves the feasibility and stability of the MTS-OPH. On the other hand, from short-distance delivery to medium-distance delivery to long-distance delivery, the number of express stations that failed to complete the delivery within the time window under the MTS-OPH changed from twelve to zero and that of the VTS-OPH changed from zero to four.

#### 5.2.4. Relationship between MTS-OPH and VTS-OPH under Different Delivery Distances

To further explore the relationship between the MTS-OPH and VTS-OPH under different delivery distances, we assumed that there was one terminal express station, did not consider the time window constraints, and quantitatively analyzed the delivery costs of the two transportation strategies.

(28) $$\begin{array}{c} f_{c}^{m}=Q*\left(2*\left(L_{ij}+L_{j^{\prime }i^{\prime }}\right)+C_{o}*\left(O_{jj^{\prime }}+2\right)\right)+2*\frac{Q}{{\text{cap}}_{V}}*C_{v}, \end{array}$$

(29) $$\begin{array}{c} f_{c}^{v}=2Q*L_{ii^{\prime }}+\frac{Q}{{\text{cap}}_{V}}*C_{v}, \end{array}$$

where *f*_*c*_^*m*^ represents the delivery cost of MTS-OPH, *f*_*c*_^*v*^ represents the delivery cost of VTS-OPH, *Q* is the number of express parcels, *C*_*v*_ is the fixed operating cost of the vehicle, cap_*V*_ is the capacity of freight vehicles, *L* is the vehicle transportation distance between two points, *C*_*o*_ is per unit cargo transfer cost after one transfer, and *O* is the number of transfers between two stations.

The delivery costs of the two transportation strategies are shown in Equations (28) and (29). Assuming that the number of express deliveries for delivery is 60 parcels that reach the upper limit of vehicle loading and the number of transfers is 2, the relationship between the two transportation strategies is

(30) $$\begin{array}{c} L_{ii^{\prime }}>1.45+\left(L_{ij}+L_{j^{\prime }i^{\prime }}\right). \end{array}$$

Equation (30) shows that when the vehicle delivery distance of VTS-OPH is greater than the sum of 1.45 and the vehicle delivery distance of MTS-OPH, the MTS-OPH should be adopted; otherwise, the VTS-OPH should be selected. The above conclusions further prove that the MTS-OPH is more suitable for long-distance delivery.

### 5.3. Impact on MTS-OPH under Different Load Rates of Passenger Flow during Off-Peak Hours

**Figure 9:**
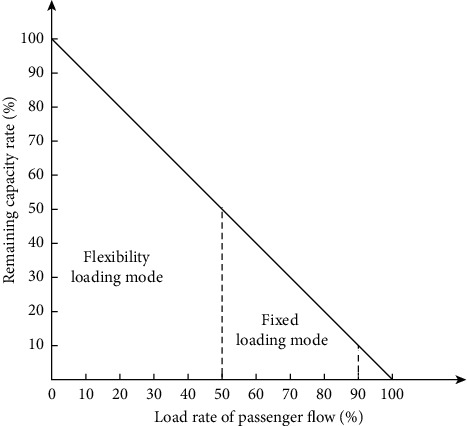
Transportation methods under different load rates of passenger flow.

In this section, we analyze the impact of different load rates of passenger flow on MTS-OPH. We selected case data in S3 for analysis, the total number of express deliveries is 975 packages, and three train carriages are required as freight carriages. This determines the 50% full-load rate during off-peak hours as the boundary. Concurrently, following the principle of passenger and cargo diversion, according to the statistics of Ningbo Rail Transit Group, the maximum passenger flow load rate of the Ningbo metro network during off-peak hours is 90% and 5 train carriages are required as passenger carriages. Therefore, 90% of the passenger flow load rate is determined as upper limit. When the full-load rate of passenger flow during the off-peak hours is greater than 90%, the MTS-OPH is not selected, as shown in Figure 9. When the interval for the full-load rate of passenger flow is [0%, 50%] and the total number of goods and passengers is less than the capacity of the metro train, the flexibility loading mode should be selected, the full-load rate of passenger flow should be (50%, 90%], the total number of goods and passengers is greater than the capacity of the metro train, and the fixed loading mode should be selected. When the full-load rate of passenger flow is greater than 90%, the metro is used for transporting passengers.

To further compare the two transportation methods in the MTS-OPH, with the load rates of passenger flow of 50% and 90% as the boundary, the VNS algorithm was run 20 times to obtain the optimal results, as shown in Table 17.

In Table 17, the delivery cost and the vehicle delivery distance are equal in Methods 1 and 2, but considering the average delivery time, Method 2 is shorter than Method 1. When the load rate of passenger flow satisfies the boundary conditions, the delivery efficiency of Method 2 is better than that of Method 1 for the same delivery time. Considering long-term transportation, MTS-OPH has the potential and has huge positive effects; for example, it can alleviate traffic congestion, has huge economies of scale [51, 52], and has much lower fuel consumption than the VTS-OPH. The MTS-OPH is adopted during off-peak hours, which can fulfill the use of the idle resources of the metro and does not require excessive initial investment.

### 5.4. Applicability of MTS-OPH considering Different Metro Networks

**Figure 10:**
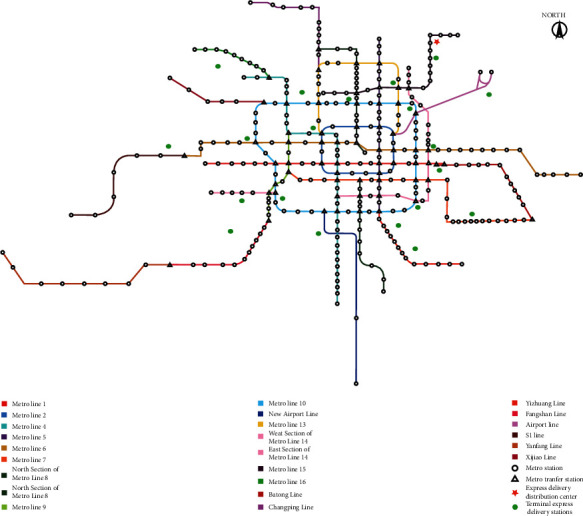
Metro network of Beijing.

In order to further prove the applicability and feasibility of the model, we selected the Beijing metro network for numerical experiments. The Beijing metro network is one of the most complex metro networks in China, with 24 metro lines totaling 331 stations, including 62 transfer stations, as shown in Figure 10. In Figure 10, the green dots indicate the terminal express delivery stations, totaling 20. The red five-pointed star indicates the express delivery distribution center, which is the airport logistics park near the capital airport. Different from Ningbo's radial metro network, Beijing's metro network is a ring-shaped radial network with more diversified route selections between lines. Therefore, we increased the maximum number of transfers in the model to 4 times.

In the experiment, the considered time horizon is set as 9:00–12:00, which is the off-peak period of metro operation. According to the data of Beijing Subway Operation Company, the maximum passenger load rates of passenger flow in the Beijing metro network during off-peak hours is 94.3%. The metro train is composed of 6 carriages, and it needs to occupy 5 carriages for passenger transportation. Thus, the loading mode of Method 1 for mixed transportation is selected. Some parameters in the experiments are set as follows. That is, the delivery time window is set to 2 hours or 3 hours, and the demand and time windows of each terminal express station are shown in Table 18. The remaining parameter values and algorithm parameter settings are consistent with the small-scale case study presented in Section 5.1.

The experimental data in Table 18 are used in the VNS algorithm for 20 calculations. The optimal solution and delivery route information are listed in Tables 19 and 20, respectively.

In Table 19, the delivery cost of MTS-OPH is 59.5% of VTS-OPH and the vehicle transportation distance is 34.1% of VTS-OPH. The average delivery time of MTS-OPH is slightly better than that of VTS-OPH, and the delivery tasks are all completed within the time window. However, under the VTS-OPH mode, there are 3 terminal express stations that failed to complete the delivery within the time window. In Table 20, terminal express stations closer to the express distribution center use VTS-OPH for shorter delivery time, while terminal express stations farther away from the express distribution center use MTS-OPH for shorter delivery time. In general, the MTS-OPH mode can complete the delivery within the time window, and the average delivery time is shorter, which is better than the VTS-OPH mode.

According to the case analysis results in Tables 19 and 20, MTS-OPH is more suitable for long-distance multitarget delivery. Compared with VTS-OPH, MTS-OPH has lower total delivery cost, shorter vehicle transportation distance, and higher service level. The above analysis results are consistent with the Ningbo metro case analysis results, which prove that our proposed model can be applied to different types of metro networks with different levels of complexity.

## 6. Conclusion

This paper proposes a new mixed transport strategy based on the metro network during off-peak hours to determine the freight mode and its distribution cost under off-peak metro passenger flow. The mixed transportation standard of passenger flow priority and separate transportation of passenger and cargo flows of the same train are proposed. According to the aforementioned criteria, a nonlinear programing model of the mixed transport strategy is constructed. In addition, an improved VNS algorithm is designed to solve the model. Finally, considering the Ningbo and Beijing metro network as an example, it is verified that the proposed model and mixed transport strategy can provide decision support for logistics companies. The main contributions of the study are as follows.

A theoretical framework of the mixed transport strategy for different metro passenger flows during off-peak hours was developed via comparisons with related studies [9, 20, 29]. Thus, a mixed transportation standard with passenger flow priority and separate transportation of the same train passenger and cargo flows was established, which expanded the integrated transportation of urban freight and metro passenger transportation.

In practice, the model of the mixed transportation strategy proposed in this study can provide decision support for logistics companies based on different delivery distances, different off-peak passenger flows, and different types of metro networks. First, when the vehicle transportation distance under separate VTS-OPH is greater than the sum of 1.45 and the vehicle transportation distance of MTS-OPH, the MTS-OPH should be adopted; otherwise, the VTS-OPH should be selected. Second, when the number of passengers and freight transportation demand are greater than the metro capacity, the fixed loading mode of the MTS-OPH should be selected for transportation; otherwise, the flexibility loading mode of the MTS-OPH should be selected for transportation. Finally, the proposed model framework can be applied to different types of metro networks with different levels of complexity.

This study has some limitations to be further solved, such as freight vehicle scheduling and route planning and metro station and line performance evaluation [53, 54]. The study should mainly focus on the following aspects: (1) Freight vehicle routes should be planned to improve vehicle utilization, and train schedule issues should be analyzed from a data-driven perspective [55]. (2) Owing to the uneven distribution of passenger flow in the urban metro system during off-peak hours, the remaining loading capacity of other carriages can be considered in future studies. (3) To ensure the mixed strategy realizable, the metro turnover and rolling stock circulation and selecting the unloading stations should be considered.

## Figures and Tables

**Algorithm 1:**
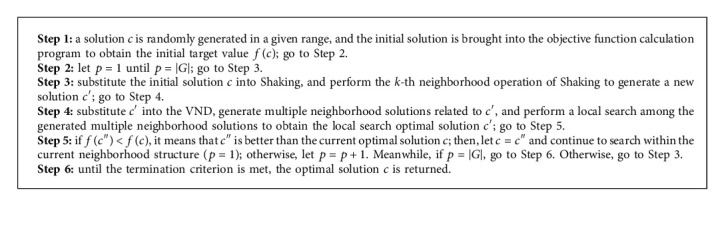
Procedure of VNS algorithm.

**Algorithm 2:**
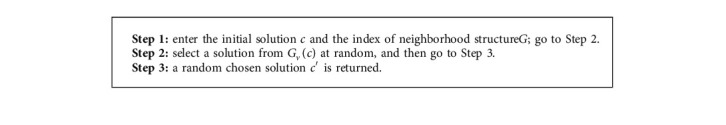
Shaking procedure.

**Algorithm 3:**
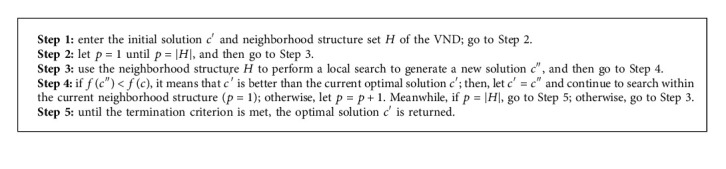
Procedure of VND.

**Table 1:** Notations.

Index sets	*J*	Set of departure metro stations, *j* ∈ *J*
	*J*′	Set of arrival metro stations, *j*′ ∈ *J*′
	*I*′	Set of terminal cargo stations, *i*′ ∈ *I*′
	*V*	Set of freight vehicles, *v* ∈ *V*
	*M*	Set of metro, *m* ∈ *M*
	*N*	Set of maximum cross-section passenger flow collection during off-peak hours, *N*_*m*_ ∈ *N*, where *N*_*m*_ is the maximum cross-section passenger flow of metro *m* during off-peak hours
Parameters	*i*	Cargo distribution center (*i*=1)
	*n*	Index of freight vehicles (*n* ∈ *V*)
	*Q*_*i*′_	Number of cargos received at terminal cargo station *i*′
	*C*_*v*_	Fixed operating cost for one vehicle
	*C*_*ij*_	Cost of per unit cargo transportation by vehicles from *i* to *j*
	*C*_*O*_	Cost of per unit cargo transfer after one transfer
	*t*_*O*_	Time taken to complete a transfer
	*D*	Number of train carriages
	*E*	Number of freight carriages
	cap_*V*_	Capacity of transport vehicles
	cap_*M*_	Freight capacity of one metro carriage
	*O*_*jj*′_	Number of transfers on metro transport path (*j*, *j*′)
	*s*_*j*_^*m*^	On the metro line where station *j* is located, the departure time of the first station of train *m*
	*t*_*j*_	Cargo loading time at station *j*
	*t*_*ij*_	Time taken to complete path (*i*, *j*)
	[ET_*i*′_.LT_*i*′_]	Receiving time window of the terminal cargo station *i*′
	*r*	1 represents Method 1 of MTS-OPH, 2 represents Method 2 of MTS-OPH
	*α*	Delay cost of unit cargo
	*λ*	0, if the delivery time is less than the latest delivery time, *α*, otherwise
	*Z*	A large positive number

Decision variables	*x*_*ij*_^*v*^	1, if the transport path (*i*, *j*) is served by the vehicle *v*, 0, otherwise
	*y*_*jj*′_^*i*′*m*^	1, if the cargo from the terminal cargo station *i*′ is delivered by the metro train *m* from the departure station *j* to the arrival station *j*′, 0, otherwise
	SET_*i*′_	Time taken to start delivery to the terminal cargo station *i*′
	SLT_*i*′_	Time taken to complete delivery to the terminal cargo station *i*′
	*K*	Quantity of transport vehicles (*K* ∈ *Z*)

**Table 2:** Values and units of certain parameters in the small-scale case study.

Parameters	Notations	Values
Vehicle fixed operating cost	*C*_*v*_	30 (yuan)
Capacity of the transport vehicle	cap_*V*_	60 (parcel)
Freight capacity of one metro carriage	cap_*M*_	330 (parcel)
Number of freight carriages	*E*	1 (carriage)
Transfer cost	*C*_*O*_	0.6 yuan/parcel
Transfer time	*t*_*O*_	6 min
Headway	*t*_interval_	7 min
The delay cost	*α*	2 yuan/parcel

**Table 3:** Express delivery station demand and time window in the small-scale case study.

Express delivery station	Express delivery station demand (parcel)	Time window (min)
A	53	[0, 60]
B	55	[0, 180]
C	59	[0, 60]
D	57	[0, 180]
E	51	[0, 180]
F	58	[0, 180]

**Table 4:** Comparison of optimization results for two algorithms.

Algorithm	Optimal calculating time (s)	Best delivery cost (yuan)	Worst delivery cost (yuan)	Average delivery cost (yuan)
VNS	86.3	5861.0	6239.8	6021.2
GA	16.2	6382.5	7618.2	6775.0

**Table 5:** Optimal solution for the two transportation strategies.

Transportation strategy	Delivery cost (yuan)	Delivery distance by vehicles (km)	Average delivery time (min)	Number of express delivery stations that did not deliver on time
MTS-OPH (Method 1)	5861.0	26.3	73	0
VTS-OPH	15350.0	136.4	69	0

**Table 6:** Delivery route comparison.

Terminal express stations	MTS-OPH (Method 1)		VTS-OPH	
	Delivery route (transfer times)	Delivery time (min)/train number	Delivery route (transfer times)	Delivery time (min)
A	I ⟶ L5I2 ⟶ L2O2 ⟶ A (1)	39/1	I ⟶ A (0)	21
B	I ⟶ L5I2 ⟶ L2O6 ⟶ B (1)	41/1	I ⟶ B (0)	20
C	I ⟶ L5I2 ⟶ L5O9 ⟶ C (0)	41/1	I ⟶ C (0)	36
D	I ⟶ L5I2 ⟶ L3O22 ⟶ C (1)	87/1	I ⟶ D (0)	75
E	I ⟶ L5I2 ⟶ L1O24 ⟶ C (1)	101/1	I ⟶ E (0)	111
F	I ⟶ L5I2 ⟶ L1O29 ⟶ C (1)	125/2	I ⟶ F (0)	147

**Table 7:** Delivery time window.

Delivery area	S1	S2	S3
Delivery time window [ET, LT] (min)	[0, 60] or [0, 90]	[0, 90] or [0, 120]	[0, 120] or [0, 180]

**Table 8:** Express delivery station demand and time window in S1.

Express delivery station	Express delivery station demand (parcel)	Time window (min)
1	56	[0, 90]
2	58	[0, 60]
3	43	[0, 90]
4	58	[0, 90]
5	53	[0, 90]
6	42	[0, 90]
7	46	[0, 90]
8	51	[0, 60]
9	59	[0, 90]
10	59	[0, 60]
11	43	[0, 90]
12	59	[0, 60]
13	59	[0, 60]
14	50	[0, 60]
15	56	[0, 60]
16	43	[0, 90]
17	48	[0, 90]
18	58	[0, 60]
19	56	[0, 90]
20	59	[0, 60]

**Table 9:** Optimal solution for two transportation strategies.

Transportation strategy	Delivery cost (yuan)	Delivery distance by vehicles (km)	Average delivery time (min)	Number of express delivery stations that did not deliver on time
MTS-OPH (Method 1)	38842.3	93.3	83	12
VTS-OPH	23349.0	214.8	33	0

**Table 10:** Delivery route comparison.

Terminal express stations	MTS-OPH (Method 1)		VTS-OPH	
	Delivery route (transfer times)	Delivery time (min)/train number	Delivery route (transfer times)	Delivery time (min)
1	I ⟶ L5I2 ⟶ L4O2 ⟶ 1 (2)	109/2	I ⟶ 1 (0)	72
2	I ⟶ L5I2 ⟶ L4O3 ⟶ 2 (2)	97/1	I ⟶ 2 (0)	57
3	I ⟶ L5I2 ⟶ L4O5 ⟶ 3 (2)	108/2	I ⟶ 3 (0)	54
4	I ⟶ L5I2 ⟶ L4O6 ⟶ 4 (2)	87/3	I ⟶ 4 (0)	57
5	I ⟶ L2I1 ⟶ L4O6 ⟶ 5 (1)	90/1	I ⟶ 5 (0)	45
6	I ⟶ L5I2 ⟶ L4O6 ⟶ 6 (2)	102/3	I ⟶ 6 (0)	42
7	I ⟶ L5I2 ⟶ L1O3 ⟶ 7 (2)	97/3	I ⟶ 7 (0)	28
8	I ⟶ L5I2 ⟶ L1O3 ⟶ 8 (2)	88/1	I ⟶ 8 (0)	23
9	I ⟶ L5I2 ⟶ L1O7 ⟶ 9 (2)	92/3	I ⟶ 9 (0)	33
10	I ⟶ L5I2 ⟶ L1O7 ⟶ 10 (2)	84/1	I ⟶ 10 (0)	22
11	I ⟶ L5I2 ⟶ L1O6 ⟶ 11 (2)	87/3	I ⟶ 11 (0)	27
12	I ⟶ L5I2 ⟶ L2O8 ⟶ 12 (1)	49/1	I ⟶ 12 (0)	19
13	I ⟶ L5I2 ⟶ L1O5 ⟶ 13 (2)	85/1	I ⟶ 13 (0)	15
14	I ⟶ L5I2 ⟶ L1O2 ⟶ 14 (2)	93/2	I ⟶ 14 (0)	11
15	I ⟶ L5I2 ⟶ L2O7 ⟶ 15 (1)	48/2	I ⟶ 15 (0)	21
16	I ⟶ L5I2 ⟶ L2O4 ⟶ 16 (1)	42/3	I ⟶ 16 (0)	18
17	I ⟶ L5I2 ⟶ L2O3 ⟶ 17 (1)	75/4	I ⟶ 17 (0)	17
18	I ⟶ L5I2 ⟶ L2O2 ⟶ 18 (1)	50/2	I ⟶ 18 (0)	20
19	I ⟶ L5I2 ⟶ L3O1 ⟶ 19 (1)	93/4	I ⟶ 19 (0)	39
20	I ⟶ L5I2 ⟶ L3O5 ⟶ 20 (1)	67/2	I ⟶ 20 (0)	29

**Table 11:** Express delivery station demand and time window in S2.

Express delivery station	Express delivery station demand (parcel)	Time window (min)
1	47	[0, 90]
2	57	[0, 90]
3	52	[0, 120]
4	51	[0, 90]
5	58	[0, 120]
6	46	[0, 90]
7	55	[0, 90]
8	55	[0, 120]
9	48	[0, 120]
10	51	[0, 120]
11	42	[0, 120]
12	41	[0, 120]
13	51	[0, 120]
14	56	[0, 120]
15	59	[0, 90]
16	43	[0, 120]
17	51	[0, 90]
18	49	[0, 90]
19	40	[0, 90]
20	47	[0, 90]

**Table 12:** Optimal solution for two transportation strategies.

Transportation strategy	Delivery cost /(yuan)	Delivery distance by vehicles (km)	Average delivery time (min)	Number of express delivery stations that did not deliver on time
MTS-OPH (Method 1)	35582.5	94.1	86	2
VTS-OPH	38661.6	378.4	57	0

**Table 13:** Delivery route comparison.

Terminal express stations	MTS-OPH (Method 1)		VTS-OPH	
	Delivery route (transfer times)	Delivery time (min)/train number	Delivery route (transfer times)	Delivery time (min)
1	I ⟶ L5I2 ⟶ L3O22 ⟶ 1 (2)	94/1	I ⟶ 1 (0)	72
2	I ⟶ L5I2 ⟶ L3O23 ⟶ 2 (2)	105/1	I ⟶ 2 (0)	86
3	I ⟶ L5I2 ⟶ L3O21 ⟶ 3 (2)	101/2	I ⟶ 3 (0)	72
4	I ⟶ L5I2 ⟶ L5O22 ⟶ 4 (1)	87/1	I ⟶ 4 (0)	69
5	I ⟶ L5I2 ⟶ L3O18 ⟶ 5 (2)	94/2	I ⟶ 5 (0)	60
6	I ⟶ L5I2 ⟶ L3O18 ⟶ 6 (2)	85/1	I ⟶ 6 (0)	45
7	I ⟶ L5I2 ⟶ L2O15 ⟶ 7 (1)	70/1	I ⟶ 7 (0)	45
8	I ⟶ L5I2 ⟶ L1O18 ⟶ 8 (1)	97/3	I ⟶ 8 (0)	69
9	I ⟶ L5I2 ⟶ L3O18 ⟶ 9 (2)	104/3	I ⟶ 9 (0)	51
10	I ⟶ L5I2 ⟶ L5O20 ⟶ 10 (1)	97/3	I ⟶ 10 (0)	72
11	I ⟶ L5I3 ⟶ L4O19 ⟶ 11 (1)	76/3	I ⟶ 11 (0)	51
12	I ⟶ L5I2 ⟶ L1O20 ⟶ 12 (1)	102/3	I ⟶ 12 (0)	60
13	I ⟶ L5I2 ⟶ L2O16 ⟶ 13 (1)	91/3	I ⟶ 13 (0)	36
14	I ⟶ L5I2 ⟶ L4O17 ⟶ 14 (1)	85/4	I ⟶ 14 (0)	39
15	I ⟶ L5I2 ⟶ L3O11 ⟶ 15 (1)	54/1	I ⟶ 15 (0)	60
16	I ⟶ L5I2 ⟶ L1O19 ⟶ 16 (1)	106/4	I ⟶ 16 (0)	39
17	I ⟶ L5I2 ⟶ L3O3 ⟶ 17 (1)	75/2	I ⟶ 17 (0)	51
18	I ⟶ L5I2 ⟶ L3O6 ⟶ 18 (1)	62/2	I ⟶ 18 (0)	57
19	I ⟶ L5I2 ⟶ L4O24 ⟶ 19 (1)	70/2	I ⟶ 19 (0)	54
20	I ⟶ L5I2 ⟶ L4O23 ⟶ 20 (1)	62/2	I ⟶ 20 (0)	48

**Table 14:** Express delivery station demand and time window in S3.

Express delivery station	Express delivery station demand (parcel)	Time window (min)
1	56	[0, 120]
2	48	[0, 120]
3	45	[0, 180]
4	48	[0, 180]
5	42	[0, 120]
6	43	[0, 120]
7	59	[0, 180]
8	59	[0, 120]
9	52	[0, 120]
10	41	[0, 120]
11	45	[0, 120]
12	47	[0, 180]
13	56	[0, 120]
14	40	[0, 120]
15	41	[0, 180]
16	43	[0, 120]
17	53	[0, 120]
18	55	[0, 180]
19	53	[0, 180]
20	49	[0, 180]

**Table 15:** Optimal solution for two transportation strategies.

Transportation strategy	Delivery cost (yuan)	Delivery distance by vehicles (km)	Average delivery time (min)	Number of express delivery stations that did not deliver on time
MTS-OPH (Method 1)	38131.8	130.8	115	0
VTS-OPH	73006.8	743.6	112	4

**Table 16:** Delivery route comparison.

Terminal express stations	MTS-OPH (Method 1)		VTS-OPH	
	Delivery route (transfer times)	Delivery time (min)/train number	Delivery route (transfer times)	Delivery time (min)
1	I ⟶ L5I2 ⟶ L2O24 ⟶ 1 (1)	105/1	I ⟶ 1 (0)	93
2	I ⟶ L5I2 ⟶ L2O26 ⟶ 2 (1)	112/1	I ⟶ 2 (0)	114
3	I ⟶ L5I2 ⟶ L2O26 ⟶ 3 (1)	127/2	I ⟶ 3 (0)	102
4	I ⟶ L5I2 ⟶ L2O24 ⟶ 4 (1)	129/3	I ⟶ 4 (0)	108
5	I ⟶ L2I1 ⟶ L2O26 ⟶ 5 (1)	112/1	I ⟶ 5 (0)	108
6	I ⟶ L5I2 ⟶ L2O21 ⟶ 6 (1)	103/1	I ⟶ 6 (0)	87
7	I ⟶ L5I2 ⟶ L1O22 ⟶ 7 (1)	118/3	I ⟶ 7 (0)	87
8	I ⟶ L5I2 ⟶ L1O21 ⟶ 8 (1)	91/1	I ⟶ 8 (0)	78
9	I ⟶ L5I2 ⟶ L1O23 ⟶ 9 (1)	96/1	I ⟶ 9 (0)	93
10	I ⟶ L5I2 ⟶ L1O27 ⟶ 10 (1)	118/2	I ⟶ 10 (0)	126
11	I ⟶ L5I2 ⟶ L1O24 ⟶ 11 (1)	101/2	I ⟶ 11 (0)	108
12	I ⟶ L5I2 ⟶ L1O25 ⟶ 12 (1)	115/3	I ⟶ 12 (0)	120
13	I ⟶ L5I2 ⟶ L2O27 ⟶ 13 (1)	119/2	I ⟶ 13 (0)	114
14	I ⟶ L5I2 ⟶ L1O26 ⟶ 14 (1)	114/2	I ⟶ 14 (0)	126
15	I ⟶ L5I2 ⟶ L1O26 ⟶ 15 (1)	127/3	I ⟶ 15 (0)	135
16	I ⟶ L5I2 ⟶ L1O26 ⟶ 16 (1)	108/2	I ⟶ 16 (0)	126
17	I ⟶ L5I2 ⟶ L1O28 ⟶ 17 (1)	116/2	I ⟶ 17 (0)	147
18	I ⟶ L5I2 ⟶ L1O28 ⟶ 18 (1)	123/3	I ⟶ 18 (0)	147
19	I ⟶ L5I2 ⟶ L1O26 ⟶ 19 (1)	128/3	I ⟶ 19 (0)	120
20	I ⟶ L5I2 ⟶ L1O22 ⟶ 20 (1)	119/4	I ⟶ 20 (0)	92

**Table 17:** Optimal solution for the two transportation methods of MTS-OPH.

Transportation method of MTS-OPH	Delivery cost (yuan)	Delivery distance by vehicles (km)	Average delivery time (min)	On-time delivery rate (%)
Method 1	37852.2	130.8	114	100
Method 2	37852.2	130.8	106	100

**Table 18:** Express delivery station demand and time window in Beijing.

Express delivery station	Express delivery station demand (parcel)	Time window (min)
1	56	[0, 120]
2	58	[0, 120]
3	43	[0, 180]
4	58	[0, 180]
5	53	[0, 120]
6	42	[0, 180]
7	46	[0, 180]
8	51	[0, 120]
9	59	[0, 120]
10	59	[0, 180]
11	43	[0, 180]
12	59	[0, 180]
13	59	[0, 120]
14	50	[0, 180]
15	56	[0, 180]
16	43	[0, 120]
17	48	[0, 180]
18	58	[0, 120]
19	56	[0, 180]
20	59	[0, 120]

**Table 19:** Optimal solution for two transportation strategies.

Transportation strategy	Delivery cost (yuan)	Delivery distance by vehicles (km)	Average delivery time (min)	Number of express delivery stations that did not deliver on time
MTS-OPH (Method 1)	49320.8	265.0	104	0
VTS-OPH	82855.2	776.0	117	3

**Table 20:** Delivery route comparison.

Terminal express stations	MTS-OPH (Method 1)		VTS-OPH	
	Delivery route (transfer times)	Delivery time (min)/train number	Delivery route (transfer times)	Delivery time (min)
1	I ⟶ L15I3 ⟶ L15O7 ⟶ 1 (0)	46/1	I ⟶ 1 (0)	27
2	I ⟶ L15I3 ⟶ L15O11 ⟶ 2 (0)	72/1	I ⟶ 2 (0)	75
3	I ⟶ LAI1 ⟶ L15O7 ⟶ 3 (1)	81/1	I ⟶ 3 (0)	45
4	I ⟶ L15I3 ⟶ L10O12 ⟶ 4 (1)	77/2	I ⟶ 4 (0)	84
5	I ⟶ L15I3 ⟶ L14O10 ⟶ 5 (1)	82/1	I ⟶ 5 (0)	91
6	I ⟶ L15I3 ⟶ L16O8 ⟶ 6 (4)	163/2	I ⟶ 6 (0)	138
7	I ⟶ L15I3 ⟶ L13O14 ⟶ 7 (1)	83/2	I ⟶ 7 (0)	112
8	I ⟶ L15I3 ⟶ L15O18 ⟶ 8 (0)	78/1	I ⟶ 8 (0)	99
9	I ⟶ L15I3 ⟶ L4O12 ⟶ 9 (2)	108/1	I ⟶ 9 (0)	106
10	I ⟶ LAI1 ⟶ L6O26 ⟶ 10 (1)	117/1	I ⟶ 10 (0)	129
11	I ⟶ L14EI1 ⟶ LS1O3 ⟶ 11 (2)	173/2	I ⟶ 11 (0)	182
12	I ⟶ L15I3 ⟶ L14EO5 ⟶ 12 (3)	164/1	I ⟶ 12 (0)	155
13	I ⟶ LAI1 ⟶ L9O11 ⟶ 13 (1)	98/3	I ⟶ 13 (0)	172
14	I ⟶ L15I3 ⟶ L1O22 ⟶ 14 (2)	28/3	I ⟶ 14 (0)	147
15	I ⟶ L15I3 ⟶ L4O24 ⟶ 15 (2)	149/1	I ⟶ 15 (0)	154
16	I ⟶ L14EI1 ⟶ L14EO16 ⟶ 16 (0)	115/1	I ⟶ 16 (0)	115
17	I ⟶ L5I3 ⟶ L2O25 ⟶ 17 (1)	155/2	I ⟶ 17 (0)	117
18	I ⟶ L15I3 ⟶ LYZO8 ⟶ 18 (2)	39/3	I ⟶ 18 (0)	143
19	I ⟶ L15I3 ⟶ L7O9 ⟶ 19 (2)	137/2	I ⟶ 19 (0)	143
20	I ⟶ L15I3 ⟶ L1O2 ⟶ 20 (2)	110/2	I ⟶ 20 (0)	102

## Data Availability

The data used to support the findings of this study are available from the corresponding author upon request.
